# A Pilot Quantitative Evaluation of Early Life Language Development in Fragile X Syndrome

**DOI:** 10.3390/brainsci9020027

**Published:** 2019-01-29

**Authors:** Debra L. Reisinger, Rebecca C. Shaffer, Ernest V. Pedapati, Kelli C. Dominick, Craig A. Erickson

**Affiliations:** 1 Division of Developmental and Behavioral Pediatrics, Cincinnati Children’s Hospital Medical Center, Cincinnati, OH 45229, USA; debra.reisinger@cchmc.org (D.L.R.); rebecca.shaffer@cchmc.org (R.C.S.); 2 Department of Pediatrics, University of Cincinnati College of Medicine, Cincinnati, OH 45267, USA; 3 Division of Child and Adolescent Psychiatry, Cincinnati Children’s Hospital Medical Center, Cincinnati, OH 45229, USA; ernest.pedapati@cchmc.org (E.V.P.); kelli.dominick@cchmc.org (K.C.D.); 4 Division of Child Neurology, Cincinnati Children’s Hospital Medical Center, Cincinnati, OH 45229, USA; 5 Department of Psychiatry and Behavioral Neuroscience, University of Cincinnati College of Medicine, Cincinnati, OH 45267, USA

**Keywords:** fragile X syndrome, language development, automated vocal analysis

## Abstract

Language delay and communication deficits are a core characteristic of the fragile X syndrome (FXS) phenotype. To date, the literature examining early language development in FXS is limited potentially due to barriers in language assessment in very young children. The present study is one of the first to examine early language development through vocal production and the language learning environment in infants and toddlers with FXS utilizing an automated vocal analysis system. Child vocalizations, conversational turns, and adult word counts in the home environment were collected and analyzed in a group of nine infants and toddlers with FXS and compared to a typically developing (TD) normative sample. Results suggest infants and toddlers with FXS are exhibiting deficits in their early language skills when compared to their chronological expectations. Despite this, when accounting for overall developmental level, their early language skills appear to be on track. Additionally, FXS caregivers utilize less vocalizations around infants and toddlers with FXS; however, additional research is needed to understand the true gap between FXS caregivers and TD caregivers. These findings provide preliminary information about the early language learning environment and support for the feasibility of utilizing an automated vocal analysis system within the FXS population that could ease data collection and further our understanding of the emergence of language development.

## 1. Introduction

Fragile X Syndrome (FXS) is the leading inherited cause of intellectual disability (ID) associated with a mutation on an unstable trinucleotide (CCG) repeat expansion on the fragile X mental retardation 1 (*FMR1*) gene [1]. FXS impacts 1 in 4,000 males and 1 in 6,000 females and, as an X linked disorder, has a more severe presentation in males. FXS is characterized by mild to severe ID with a series of other features including: anxiety, social deficits, communication deficits, gaze aversion, inattention, impulsivity, aggression and hyperactivity [2]. Within communication deficits, it is evident in the current literature that FXS is associated with significant language delay, above that expected by given cognitive deficits, with relevant strengths in receptive communication and relative weaknesses in expressive communication [3,4]. Unfortunately, it can be quite challenging to accurately assess early language acquisition in infants and young children due to the natural development of language. This can be particularly difficult in clinical populations with known speech delays (e.g., FXS, autism spectrum disorder, Down syndrome) potentially impacting early diagnostic and treatment efforts. 

Within the typically developing population, infants can perceive and attend to speech in comparison to silence or other sounds prior to speaking their first word [5,6]. The progression of expressive language development has universally been identified as cooing (between 1 and 4 months), to babbling (between 5 and 10 months), to meaningful speech (between 10 and 18 months) [7]. The social environment and interactions with caregivers throughout infancy and toddlerhood provide key building blocks for language development [8,9]. Specifically, the amount of language in a child’s environment prior to the age of three is significantly correlated with language acquisition and cognitive development [10,11]. Furthermore, differences in early language development (e.g., use of babbling, frequency of vocalizations) have been found to differentiate infants with atypical development and typical development including infants with autism spectrum disorder (ASD) [12,13], Williams Syndrome [14], and FXS [15]. 

Prospectively in the ASD literature, infants with an older sibling diagnosed with ASD who later went on to have their own diagnosis of ASD demonstrated significant declines in their trajectories of receptive and expressive communication across 6 to 36 months of age [13]. Retroactively through home videos, infants later diagnosed with ASD have been shown to exhibit reduced canonical babbling and fewer vocalizations deemed relevant for the development of speech across 9 to 15 months of age [12]. Unfortunately, neither of these studies took into consideration the impact of cognitive development on their language development. Research examining language development in toddlers with ASD have shown a discrepancy between language abilities and their nonverbal cognitive level suggesting that these language deficits exist in this population despite their cognitive abilities [16]. Similar findings have also been observed in infants with Williams syndrome, suggesting overall delays in first word production and canonical babbling [14] despite their relative strengths in language in adolescence and adulthood. 

Communication deficits in school-aged children and adolescents with FXS have been investigated extensively in the literature [3,4,17,18,19]. Individuals with FXS have reported deficits across all aspects of language (e.g., comprehension, pragmatics, expressive and receptive skills) with these deficits remaining throughout life into adulthood. Unfortunately, the literature assessing language development in infancy and toddlerhood is limited. Roberts, Hatton, and Bailey (2001) [20] reported the age in which infants with FXS spoke their first word was delayed by approximately 17 months; however, considerable variability was noted in their sample with 30% of the infants with FXS speaking their first word within age-expected limits. Similar findings were observed by Hinton et al. (2013) [21] where infants with FXS spoke their first word around 26.2 months. Two studies have utilized retrospective home videos to examine communication abilities of infants with FXS between the ages of 9 and 12 months [15,22]. Marschik et al. (2014) [22] utilized the Inventory of Potential Communicative Acts (IPCA) [23] with seven children with FXS to assess social-communicative forms and functions where specific deficits were identified in requesting, imitating, and decision making. Belardi et al. (2017) [15] utilized a naturalistic listening approach to identify deficits in canonical babbling (e.g., producing adult-like syllables) and the frequency of vocalizations in infants with FXS. Utilizing standardized assessments and parental report for language development to assess how visual attention at 12 and 18 months impacts language outcomes, Kover et al. (2015) [24] found that infants with FXS were significantly delayed based on both chronological and developmental expectations of language ability. Furthermore, the infants with FXS were found to acquire language at a slower rate than their chronological expectations and are likely to fall further behind over time. Overall, infants with FXS are reportedly exhibiting notable delays in their language abilities early on in development; however, the current literature lacks prospective, quantitative yet naturalistic methodologies to assess the emergence and development of these language deficits during the earliest periods of development. 

Examining the language learning environment of young children, in particular their social interactions with caregivers, also provides insight into their language development. [8,9]. Within the ASD literature, Warren et al. (2010) [25] found that young children with ASD engaged in fewer caregiver interactions and vocalizations than typically developing children. They also demonstrated that their vocal productions increase as the number of words that are addressed to them increases. Within the FXS literature, little research exists examining their social or language environment and how this impacts language development. Drawing on the recent work examining maternal responsivity and language development in young children with FXS, low levels of maternal responsivity have been found to be related to deficits in receptive and expressive communication abilities along with vocabulary development in FXS [26,27]. Interestingly, the rate of child communication has been found to significantly negatively impact maternal responsivity [28] suggesting a disrupted cycle of both children with FXS and their caregivers communicating less. Further, the literature examining maternal responsivity in FXS has primarily utilized short structured activities and brief naturalistic observation to assess child language development through effortful, behavioral coding procedures. The potential ability to assess the language environment, child language abilities, and caregiver vocalizations in their natural environment through an efficient manner for longer time periods utilizing a noninvasive approach would further our current understanding of early language development in FXS.

The present study aims to build on our current knowledge of early language development in FXS while addressing some of the challenges to assessment in very young children. Utilizing a pilot sample of infants and toddlers with FXS, the present study examines child and caregiver vocalizations in their home environment utilizing an automated vocal analysis system. Consistent with the literature described above, we hypothesize that the infants with FXS will be below their chronological and developmental age expectations for vocalization use in comparison to age-matched typically developing peers. Furthermore, we hypothesize that the caregivers of the infants and toddlers with FXS will also utilize less vocalizations in comparison to other caregivers with typically developing children. Additional exploratory analyses were assessed for potential relationships between parent vocalizations and child vocalizations in the FXS sample. This preliminary study is the first to assess the utility of a noninvasive automated vocal analysis system in individuals with FXS. 

## 2. Method

### 2.1. Participants

Eleven males with a confirmed molecular diagnosis of full mutation FXS between the ages of 17 to 64 months of age (*M* = 41.58, *SD* = 13.43) participated in the present pilot study. Data were drawn from a longitudinal study at Cincinnati Children’s Hospital Medical Center as a subcomponent of a larger, multi-site study developing a nationwide research database in FXS. Cincinnati Children’s Hospital Medical Center Institutional Review Board (IRB) approved the study protocol (IRB #: 2012-2445) and caregivers signed informed consent for their children to participate. Data were extracted from the LENA Foundation Natural Language Study [29] to derive a typically developing (TD) normative dataset to compare to the performance of the children with FXS. Comprehensive results for that study are reported in Gilkerson and Richards (2008) [29]. Two samples of TD normative data were utilized to match the FXS sample by both chronological and developmental age. The developmental age of the FXS sample ranged between 6 and 22 months of age (*M* = 14.67, *SD* = 5.10). The final FXS sample resulted in nine males between the ages of 17 and 58 months (*M* = 38.33, *SD* = 13.05) after excluding two participants (see details below under “LENA”).

### 2.2. Measures

#### 2.2.1. LENA

The LENA system includes a digital language processor (DLP) that is worn by the participant and a language analysis software. The DLP is a small digital recorder that is worn in a specially designed child’s shirt. The device continually records the child’s vocalizations and the language environment within a four to six foot radius around the child for up to 16+ hours. Once the recording is completed, an audio file from the DLP is transferred to a computer and processed by the LENA language analysis software. The software provides data for three main variables: child vocalizations (frequency and duration), adult word count, and conversational turns. The device also provides data for other variables in the environment including: TV/Electronics, Noise (e.g., bumps/rattles), Distant Sounds, Silence/Background Noise, and Overlapping Speech. Each participants’ first LENA analysis data point was extracted for the current analysis. One participant was removed from the dataset due to extreme outlier findings across all variables reported. This participant was 42 months of age with a developmental age of 36 months with more than double the amount of vocalizations and conversational turns in comparison to the rest of the sample, causing the FXS sample to be skewed. Another participant was removed for only having one hour of data collected.

For the purpose of this study, we chose to focus on the three main variables provided by the LENA system. Child vocalizations (CV) included words, babbles, and pre-speech communicative sounds. Adult word count (AWC) is an estimate of the number of words spoken near the child. A normative value for average AWC was derived from the LENA Foundation Natural Language Study [29] in order to compare the FXS AWC sample to the normal population. Specifically, we utilized the AWC at the 50th percentile. Conversational turns (CT) in the LENA output occur when a child vocalizes and an adult responds, or an adult speaks and the child responds. The reliability and validity of the LENA automated vocal analysis system has been extensively researched in the literature to examine the automated vocalization systems ability to accurately label the recorded vocalizations correctly. Xu et al. (2008) [30] reported in comparison to the transcribers’ labeling, the automated system correctly identified 82% of the segments transcribers labeled as Adult Speech and 76% of the segments labeled as Child Vocalizations. Further, adult word count estimates were on average 98% accurate compared to human transcribers’ word counts over a 12 hour recording day. Other groups have also found adequate correlations between human coders and the LENA system ranging between 0.71 and 0.85 [31] providing additional support for the accuracy of the LENA automated vocal analysis system.

A recording was considered valid if it contained at least two hours of data. As mentioned above, one participant was dropped due to having only one hour of data. The two hour criteria was established as an attainable goal for our families given the sensory challenges in the FXS population and whether or not wearing the device would be tolerable. The amount of data collected in the remaining nine participants ranged from 7 to 18 hours (*M* = 12.56, *SD* = 3.81). Since the TD normative data was derived from the LENA Foundation Natural Language Study [29] and the chronological age range for their typical sample was between 2 and 48 months, developmentally age-matched norms were extracted for all of the FXS participants; however, two participants were outside of the 48 month window chronologically and therefore not included in the chronological age-matched analyses. 

#### 2.2.2. LENA Developmental Snapshot (LDS)

The LENA Developmental Snapshot [32] is a caregiver-report questionnaire that assesses both receptive and expressive language skills for children ages 2 to 36 months of age. The LDS consist of 52 items answered with a “yes” or “not yet” about the child’s behavior (e.g., “Does your child vocalize while gesturing to let you know what he/she wants?”). Domains within the questionnaire focus on vocal behavior and preverbal communication for infants under 12 months; responsiveness to instruction, spontaneous speech production and vocabulary development for 1-year-olds; and conceptual and grammatical development for children over 24 months [33]. The LDS is scored automatically online through the LENA Online system. The number of “yes” answers reported are added up to create a total raw score which is then transformed into a Developmental Age. The LDS was found to be highly correlated (0.81–0.97) with other widely used standardized language development assessments [32]. The Developmental Age was extracted from the questionnaire for the FXS participants and used to create a developmentally age-matched TD comparison sample based on the TD data provided in LENA Foundation Natural Language Study [29]. For example, if a child with FXS had a Developmental Age of 14 months, the 14 month data points for CV and CT from the TD sample through the Natural Language Study were extracted. 

### 2.3. Procedures

Following completion of guardian informed consent, participants’ caregivers were given in-person or mailed the LENA device along with the appropriate LENA-specific clothing to hold the device. Instructions were included on how to turn on the device and start the recording. Caregivers were instructed to have the participant wear the device during a normal day for them (e.g., avoid when they are sick or attending loud events). Additionally, they were instructed to have the participant wear the LENA clothing with the device for the entire day with the exception of taking a bath or naps; however, the device should still be nearby during these activities. The LDS was also included with the LENA device and the caregivers were asked to complete the form prior to the return of the device. Once completing the form and recording, the families were provided with materials to mail the device and questionnaire back. Once returned, the audio file was downloaded from the LENA device and uploaded to the LENA language analysis software to extract the data and the LDS was entered into the LENA online scoring system. 

### 2.4. Data Analysis

Analyses were conducted in R version 3.5.1 (R Foundation for Statistical Computing, Vienna, Austria). First, data were examined for outliers, nonnormality, and homoscedasticity. One participant with FXS was found to be a significant outlier across all variables and was removed from the analyses. Data collected on the LENA device were all converted to hourly values in order to account for the variability in duration of data collection within and across groups. In order to analyze the differences between the FXS sample and TD infants in regard to their early language development, independent-sample *t*-tests and one sample *t*-tests were conducted. The first set of independent *t*-tests examined the FXS sample in comparison to their chronologically age-matched TD peers for CV and CT. The second set of independent *t*-tests examined the FXS sample in comparison to their developmentally age-matched TD peers for CV and CT. Next, a one sample *t*-test was conducted to compare the AWC of the FXS sample to the TD average AWC at the 50th percentile. Lastly, exploratory correlational analyses were conducted to examine relationships between AWC and the other LENA variables (CV and CT) within the FXS sample. 

## 3. Results

### 3.1. Child Vocalizations

#### 3.1.1. Chronological Age Comparisons

**Figure 1:**
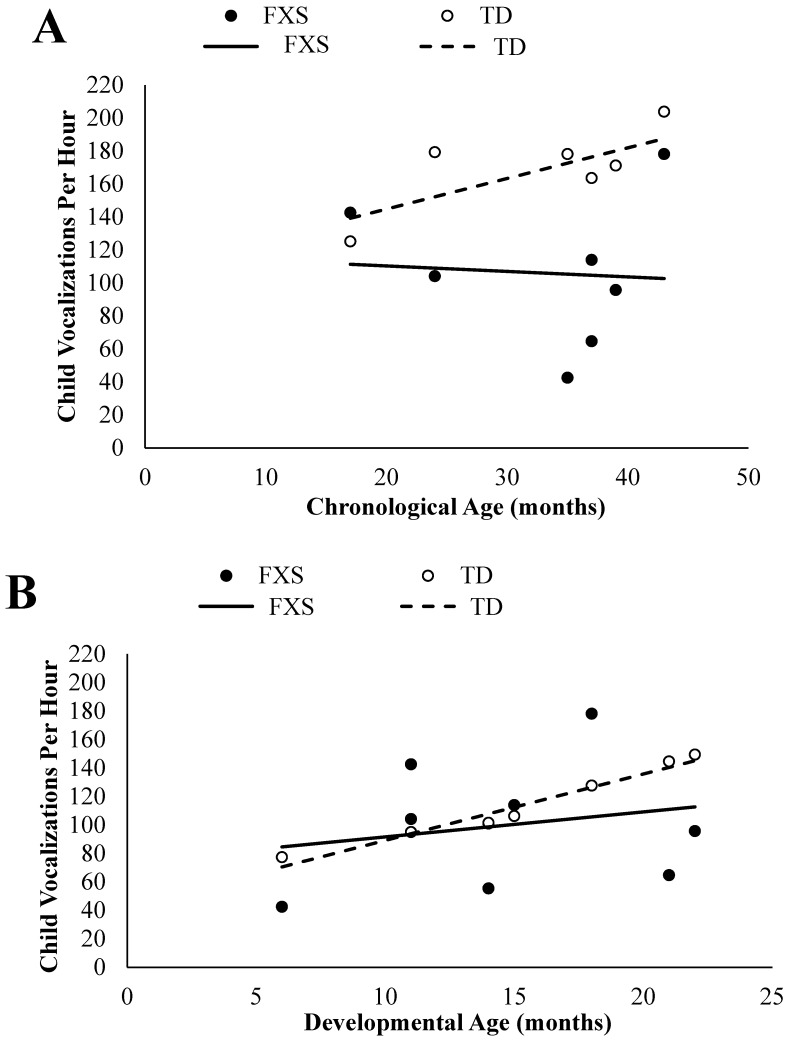
Infants and toddlers with fragile X syndrome (FXS) vocalizations per hour plotted in comparison to their chronologically age-matched (**A**) and developmentally age-matched (**B**) typically developing (TD) peers with trend lines.

An independent samples *t*-test was conducted in order to determine if infants and toddlers with FXS differed significantly in the frequency of their vocalizations in comparison to chronologically age-matched TD peers. Significant group differences were found, *t*(12) = −3.26, *p* = 0.007, *d* = 1.74. Specifically, infants and toddlers with FXS (*M* = 106.00, *SD* = 45.56) had significantly less vocalizations on average per hour than their chronologically age-matched TD peers (*M* = 169.31, *SD* = 23.73). In Figure 1A, each participant with FXS’s frequency of vocalizations are graphed in comparison to their chronologically age-matched TD peers. 

#### 3.1.2. Developmental Age Comparisons

An independent samples *t*-test was conducted in order to determine if infants and toddlers with FXS differed significantly in the frequency of their vocalizations in comparison to developmentally age-matched TD peers. No significant group differences emerged, *t*(16) = −0.68, *p* = 0.507, *d* = 0.32. The infants and toddlers with FXS (*M* = 99.84, *SD* = 42.86) had similar average vocalization frequencies to their developmentally age-matched TD peers (*M* = 110.99, *SD* = 24.32) per hour. In Figure 1B, each participant with FXS’s frequency of vocalizations are graphed in comparison to their developmentally age-matched TD peers.

### 3.2. Conversational Turns

#### 3.2.1. Chronological Age Comparisons

**Figure 2:**
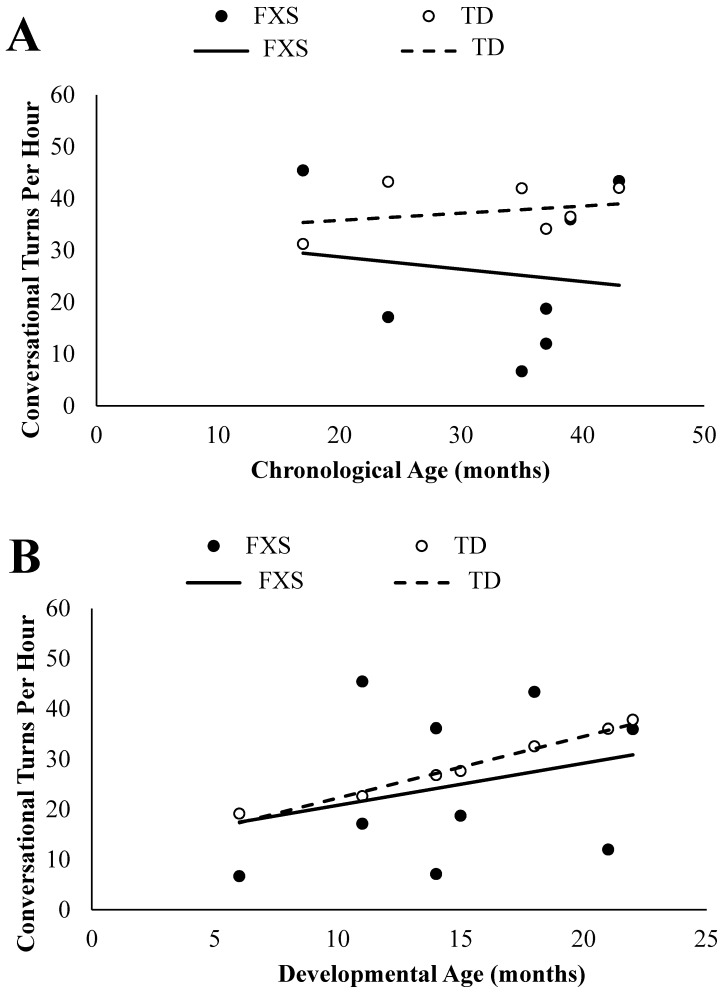
Infants and toddlers with fragile X syndrome (FXS) conversational turns per hour plotted in comparison to their chronologically age-matched (**A**) and developmentally age-matched (**B**) typically developing (TD) peers with trend lines.

An independent samples *t*-test was conducted in order to determine if infants and toddlers with FXS differed significantly in the frequency of their conversational turns with caregivers in comparison to chronologically age-matched TD peers. Levene’s test for equality of variances indicated unequal variances between groups (*F* = 20.98, *p* = 0.001), so the degrees of freedom were adjusted from 12 to 7. Marginally significant group differences were found, *t*(7) = −1.93, *p* = 0.094, *d* = 1.03. The infants and toddlers with FXS (*M* = 25.63, *SD* = 15.71) had less conversational turns per hour with their caregivers than their chronologically age-matched TD peers (*M* = 37.63, *SD* = 4.77). In Figure 2A, each participant with FXS’s frequency of conversational turns are graphed in comparison to their chronologically age-matched TD peers. 

#### 3.2.2. Developmental Age Comparisons

An independent samples *t*-test was conducted in order to determine if infants and toddlers with FXS differed significantly in the frequency of their conversational turns with caregivers in comparison to developmentally age-matched TD peers. Levene’s test for equality of variances indicated unequal variances between groups (*F* = 17.77, *p* = 0.001), so the degrees of freedom were adjusted from 16 to 11. No significant group differences emerged, *t*(11) = −0.59, *p* = 0.568, *d* = 0.28. The infants and toddlers with FXS (*M* = 24.75, *SD* = 15.52) had similar average rates of conversational turns per hour with their caregivers to their developmentally age-matched TD peers (*M* = 28.04, *SD* = 6.33). In Figure 2B, each participant with FXS’s frequency of conversational turns are graphed in comparison to their developmentally age-matched TD peers.

### 3.3. Adult Word Count

**Figure 3:**
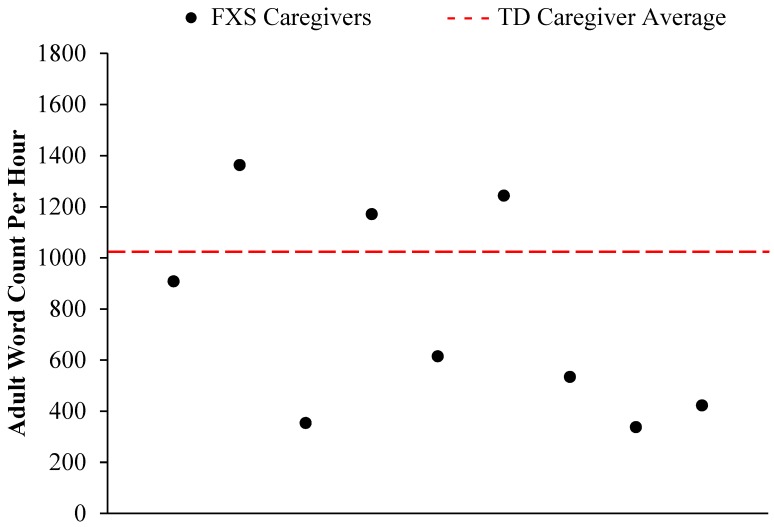
Caregivers’ of infants and toddlers with FXS adult word count per hour plotted in comparison to the average adult word count per hour in TD caregivers (*M* = 1024.75).

A single sample *t*-test was conducted to determine if a statistically significant difference existed between the FXS caregivers’ and the TD caregivers’ word count per hour. Results suggest that FXS caregivers (*M* = 772.04, *SD* = 405.75) had marginally significantly different word counts per hour in comparison to the TD caregivers (*M* = 1024.75, *t*(8) = −1.87, *p* = 0.099, *d* = 0.60. In Figure 3, each caregiver’s word count is graphed in comparison to the average adult word count for TD caregivers. 

Exploratory correlations were utilized to examine relationships between the AWC and the other LENA variables (CV and CT). Significant associations between AWC and CV (*r* = 0.82, *p* = 0.025) as well as AWC and CT (*r* = 0.98, *p* = 0.000) emerged. 

## 4. Discussion

### 4.1. Summary of Findings

Language deficits are core characteristics of the FXS phenotype. Previous literature has identified deficits in receptive and expressive communication abilities as early as 9 months of age in FXS [15,22] with their first word being vocalized between 26 and 28 months [20,21] and a reciprocal negative relationship between child vocalizations and maternal responsivity on language development and acquisition [26,27]. The present preliminary study examined early language development through frequency of child vocalizations, conversational turns between caregivers and child, and adult vocalizations in infants and toddlers with FXS in comparison to a chronologically and developmentally age-matched typically developing sample. This pilot study is one of the first to utilize an automated vocal analysis program within the FXS population. 

Partially aligning with our hypotheses and previous literature [15,22,24], infants and toddlers with FXS were found to vocalize less and engage in fewer conversational turns with their caregivers in comparison to chronologically age-matched TD peers. Despite previous research suggesting otherwise [4,24], differences in the frequency of vocalizations and conversational turns were not observed when compared to a developmentally age-matched TD group in our pilot sample. This could be explained by the differences in language assessment methodology. Specifically, majority of the literature assessing language development in infants and toddlers with FXS have utilized standardized measures obtaining norm referenced scores rather than the actual frequency of vocalizations in their normal environment. Given the known cognitive delays and receptive deficits in FXS, it can be complicated to obtain an accurate score representing their true language abilities utilizing standardized measures in the youngest children with FXS. Utilizing the LENA device allowed the present study to automatically and noninvasively obtain a naturalistic language/vocalization sample in the child’s normal environment without limitations imparted by cognitive level or receptive communication deficits, which are known to impact language abilities in FXS [34]. In sum, our preliminary results support the current body of literature across the FXS lifespan suggesting deficits in verbal communication development; however, these deficits may be accounted for by their developmental level and additional research is needed to support these findings. Further, the LENA device may be a potential new mechanism for assessment of not only language but also the language environment in FXS. 

Consistent with our hypotheses and previous literature [26,27], our results suggest caregivers of infants and toddlers with FXS produced fewer vocalizations around their children in comparison to caregivers with TD infants and toddlers. Despite the small sample size of this preliminary study, moderate effect sizes were still reported. These findings of reduced adult vocalizations coupled with reduced conversational turns between caregivers and their infants and toddlers with FXS are the first to provide insight into the language environment within FXS. Furthermore, a strong positive association between caregiver vocalizations and child vocalizations emerged suggesting that for the FXS caregivers who vocalized more, their children also vocalized more. These findings align with concerns for a potentially disrupted cycle of communication to evolve between caregiver and child in regard to the frequency of vocalizations in their language learning environment. Specifically, difficulty could arise for caregivers to maintain their frequency of vocalizations when their children are less responsive, which can unfortunately create a cycle of reduced communication across both groups potentially impacting language development for the child. Further work is needed to assess the impact of caregiver vocalizations and conversational turns between caregivers and their children with FXS on child vocalizations to delineate this hypothesis and to determine whether a true gap exists for FXS caregiver vocalizations in comparison to TD caregivers. Nevertheless, there is an existing body of literature demonstrating that caregivers can learn to be more responsive resulting in positive language outcomes for their children [35,36,37]. As for the FXS literature, there is a promising emerging body of intervention research demonstrating increases in maternal verbal responses and child prompted communication [38]. Therefore, there is hope in changing caregiver behavior through appropriate and effective interventions that can potentially close this communication gap early on, while positively impacting their child’s language outcomes and potentially their overall quality of life. 

### 4.2. Limitations

A primary limitation of this preliminary pilot study is its small sample size. Despite this, the sample is similar to those of other studies examining language development in infants and toddlers with FXS [15,21,23]. Additionally, the present study did not contain its own typically developing matched control sample to compare the FXS population too; however, utilizing the LENA Natural Language Study [29] was also a strength by allowing for the present study to utilize a more accurate, large-scale normed TD sample. Furthermore, the present study utilized a questionnaire to assess developmental level, which relies on parental report, rather than a standardized test administered by a clinician. These analyses were also limited to utilizing a cross-sectional design with one day of language data per child. Since language production can vary from day to day in infants and toddlers, it would be ideal to have more than one day of language data available. Lastly, reliability of the LENA device could be assessed through pairing human coding and the automated vocal analysis to determine the accuracy of the system specific to FXS. 

## 5. Conclusions and Future Directions

The present study aimed to build on our current understanding of early language development in FXS utilizing new methodology. Our results suggest that communication deficits, particularly vocalization production deficits, are apparent very early on in development in comparison to chronological age expectations. However, language profiles in FXS as measured by LENA appear to potentially be in line with their developmental expectations. Additional work is needed to replicate these findings using the same methodology with a larger sample and wider age range. Utilizing a longitudinal design to obtain a more accurate assessment of language development would be ideal to further our understanding of the rate of growth in language across development. Furthermore, initial details about the language learning environment for infants and toddlers with FXS were examined with additional evidence emerging for a potentially disrupted cycle of communication between FXS caregivers and their children with reduced caregiver vocalizations being associated with reduced child vocalizations. Future studies should continue to assess the effectiveness of interventions for FXS caregivers to increase their responsiveness and vocalizations on child language outcomes. Lastly, the methodology utilized in the present study provided a measure of communication abilities in infants and toddlers with FXS and insight into the language learning environment that was noninvasive and easy to use for their families. This methodology may be promising for future researchers, the participants, and their families by simplifying data collection without reducing quality and accuracy. The LENA device may continue to be utilized in future FXS research to not only quantify vocal production development and the language learning environment, but also assist in collecting outcome data for future intervention studies.
